# Experience with Bloodborne Pathogen Exposures Among Operation Room Nurses in Korea: A Qualitative Study

**DOI:** 10.1017/ash.2021.141

**Published:** 2021-07-29

**Authors:** JaHyun Kang, Hyunsook Lee

## Abstract

**Background:** Operation room (OR) nurses are at a high risk for bloodborne pathogen (BBP) exposures because they are constantly in contact with blood and body fluid (BBF) through surgical sites, and they frequently use sharp surgical instruments and sutures during surgeries. We explored occupational experiences with BBP exposures among OR nurses in South Korea. **Methods:** With an institutional review board’s approval, this qualitative research was performed with 12 OR nurses who had worked for >3 months and had experienced BBP exposures. Based on the main research question “How is the experience of BBP exposure among OR nurses?” the semistructured questions for in-depth interview were prepared. Narrative data were collected through 1-hour individual interviews from June to September 2020 and were analyzed using a thematical analysis method. **Results:** The average age of the participants was 34.4 years with an average 9.75 years of clinical experience. The main theme extracted was “The nurses are alerted to their safety after experiencing the aftereffects and emotional trauma from BBP exposures,” with 4 subthemes and 14 concepts. The first subtheme, “OR nurses risking exposure to BBF,” included (1) hurried doctors and hasty nurses; (2) sharp surgical instruments everywhere; (3) deprioritized self-protection due to ongoing surgery; (4) inattentive to BBF risk; and (5) uncomfortable, foggy goggles and receded safety devices. The second subtheme, “BBP exposures occurred in a flash,” included (1) sharp injury occurred in a split second; (2) temporarily treat sharp wounds while hiding frightened feelings; and (3) OR nurses concentrated on surgery by suppressing anxiety and sharp wounds. The third subtheme, “Burdened time that could be overwhelmed alone,” included (1) BBP exposures moments that I wish to reverse; (2) anger over dangerous environments and the turmoil of anxiety; (3) exhausted body and facial discoloration due to taking postexposure prophylaxis; and (4) exposure to BBP that I want to hide from family and friends. The fourth subtheme, “Voice for everyone’s safety,” included (1) establishing a safety culture, which requires everyone’s efforts, and (2) necessity of practical resources for decreasing BBP exposures. **Conclusions:** The participating OR nurses felt that they were working with a high risk to BBF exposures and called for institutional interventions to reduce the risks, including surgeons’ attentive collaboration. Korean hospitals should make greater efforts to establish safety culture in ORs and to provide repeated, tailored education to prevent BBF exposures. They should also supply high-quality protective equipment and safety-engineered devices for OR nurses.

**Funding:** No

**Disclosures:** None

